# High Histological Skeletonization as a Measure of Heterogeneity Is Associated with Poor Overall Survival and Death Within 2 Years of Diffuse Large B-Cell Lymphoma

**DOI:** 10.3390/cancers18172828

**Published:** 2026-09-01

**Authors:** Joaquim Carreras, Yara Yukie Kikuti, Shunsuke Nagase, Giovanna Roncador, Haruka Ikoma, Atsushi Ito, Makoto Orita, Sakura Tomita, Yuki Tanigaki, Akihisa Ueno, Yusuke Kondo, Naoya Nakamura, Yohei Masugi

**Affiliations:** 1 Department of Pathology, School of Medicine, Tokai University, 143 Shimokasuya, Isehara 259-1193, Japan; yy-kikuti@tokai.ac.jp (Y.Y.K.); nagase@tokai.ac.jp (S.N.); ih3822@tokai.ac.jp (H.I.); ito.atsushi.s@tokai.ac.jp (A.I.); orita.makoto.k@tokai.ac.jp (M.O.); sakura.t@tokai.ac.jp (S.T.); tanigaki.yuki.s@tokai.ac.jp (Y.T.); akihisaueno@tokai.ac.jp (A.U.); ky2948@tokai.ac.jp (Y.K.); naoya@tokai.ac.jp (N.N.); masugi@tokai.ac.jp (Y.M.); 2 Monoclonal Antibodies Unit, Spanish National Cancer Research Center (CNIO), Centro Nacional de Investigaciones Oncologicas, C/Melchor Fernandez Almagro, 3, 28029 Madrid, Spain; groncador@cnio.es

**Keywords:** diffuse large B-cell lymphoma, lymphoma, large B-Cell, diffuse, entropy, computer vision, skeletonization, prognosis, gene expression, immuno-oncology, survival

## Abstract

Background: Diffuse large B-cell lymphoma (DLBCL) is an aggressive lymphoma that is histologically and clinically heterogeneous. Skeletonization is a computer vision technique used in image description, segmentation, and pattern recognition. Objectives: This retrospective observational study analyzed the 2D-topological heterogeneity of DLBCL. Methods: A series of 153 patients, including 110 patients with DLBCL and 43 patients with reactive lymphoid tissue, were analyzed by skeleton analysis using hematoxylin and eosin (H&E) digitized images. The skeletonization variable was correlated with several clinicopathological characteristics of the patients, including cell of origin Hans’ classifier and Lymph2Cx assay, MYC and BCL2 rearrangement by FISH, immunohistochemistry of BCL2, CASP8, cCASP3, CD163, CDK6, cPARP, CSF1R, E2F1, ISY1, Ki69, LMO2, MDM2, MYC, MYO, TNFAIP8, PD-L1, and p53, and gene expression using a pancancer immune profiling panel. Results: In comparison with reactive tissue, DLBCL was characterized by lower skeletonization: 5665.20 ± 1012.82 vs. 6341.16 ± 548.23, respectively (*p* < 0.001). Within the DLBCL diagnostic category, high skeletonization correlated with poor overall survival (hazard risk = 2.5, *p* = 0.003). High skeletonization was also correlated with higher Epstein–Barr virus (EBV) EBER positivity, death within the first two years, high histological entropy, and lower CD5, E2F1, BCL2, ISY1, and TNFAIP8 protein levels (all *p* values < 0.05). Gene expression was available in a representative subset of 30 cases, including 18 cases with high skeletonization and 12 cases with low skeletonization. High skeletonization was characterized by upregulation of 166 immuno-oncology genes, such as CD274 (PD-L1), NFKB1A, CD68, CSF1R, CCR5, ITGAM, and TGFB1, and enrichment of IL6 JAK STAT3 signaling, inflammatory response, TNF, NFKB, KRAS, apoptosis, and macrophage function pathways by gene set enrichment analysis (GSEA). In multivariate COX regression analysis for overall survival, the prediction value of skeletonization was independent of Hans’ classifier, the International Prognostic Index (IPI), and EBER (*p* = 0.030, hazard risk = 2.2). Conclusions: DLBCL is characterized by lower skeletonization than reactive lymphoid tissue. In DLBCL, high skeletonization is associated with poor prognosis and enrichment of immuno-oncology markers.

## Simple Summary

Diffuse large B-cell lymphoma (DLBCL) is an aggressive lymphoma characterized by a diffuse proliferation of large neoplastic B lymphocytes. DLBCL is histologically and clinically heterogeneous. Skeletonization is a useful computer vision technique for feature extraction and represents an object’s topology. This study used the skeletonization function to analyze the topological heterogeneity of DLBCL in a series of 153 patients, including 110 patients with DLBCL and 43 patients with reactive lymphoid tissue. DLBCL was characterized by lower skeletonization than reactive lymphoid tissue. In DLBCL, high skeletonization was associated with poor prognosis and enrichment of immuno-oncology markers.

## 1. Introduction

Diffuse large B-cell lymphoma (DLBCL) is one of the most frequent lymphomas, accounting for approximately 25% of non-Hodgkin lymphomas [1].

DLBCL usually presents as a symptomatic lymph node enlargement in the neck or trunk, but extranodal involvement is also possible. In 30% of the cases, the patients manifest fever, night sweats, or weight loss, known as “B” symptoms [1]. The incidence of DLBCL is around 3–7 cases per 100,000 persons per year [2].

At presentation, in around 60% of the patients, the disease presents as advanced stage III-IV and elevated lactate dehydrogenase (LDH) in serum. Bone marrow is infiltrated in up to 30% of the cases. In the case of nodal DLBCL, secondary involvement of other organs such as the liver, kidney, lung, bone marrow, and central nervous system can occur [3,4,5,6].

From a histopathological point of view, DLBCL is characterized by a diffuse proliferation of large transformed B lymphocytes with prominent nucleoli and basophilic cytoplasm with abundant proliferation. The neoplastic lymphocytes resemble centroblasts or immunoblasts, and other morphologies, such as anaplastic, multinucleated, clear-cell, epithelioid, and spindle-cell, can be found [6,7,8,9,10,11].

At diagnosis, immunohistochemistry and/or flow cytometry are used to determine the immunophenotype at the protein level, which includes B-cell markers (CD20, CD79a), CD10, BCL6, and MUM1 (IRF4), for assessing the cell-of-origin Hans’ classification, and BCL2, Ki67, MYC, CD5, and p53, for evaluating the proliferation status and prognostication [12,13,14,15,16,17,18,19]. Both the immunophenotype and cytological morphologies are useful in DLBCL diagnosis and prognosis [20].

Cytogenetic features are also analyzed despite no typical or diagnostic change. *MYC*, *BCL2*, and *BCL6* gene rearrangements are routinely used to subclassify DLBCL into the DLBCL/high-grade B-cell lymphoma categories associated with a more aggressive clinical evolution [21,22,23,24,25,26,27,28,29,30].

The prediction of DLBCL prognosis using artificial intelligence and digital pathology has gained interest recently. For example, Jeong Honn Lee et al. recently retrospectively collected 251 slide images and performed deep learning and multi-modal prediction with contrastive learning and feature extraction that provided a performance with an area under the curve of 0.8 in the ROC analysis [31]. Other recent works include the work of Vrabac D et al. [32], Li Z. et al. [33], and Ma D. et al. [34] that also focused on the evaluation of histological images. However, the focus on topological features is less studied.

Topology is the study of the properties of geometric figures or spaces [35]. In mathematics, low-dimensional topology focuses on the study of manifolds or topological spaces of four or fewer dimensions [36]. Originating from the studies of two-dimensional states, we developed the concept of histological complexity in cancer. In applied mathematics, the extraction of information from high-dimensional, incomplete, and noisy datasets is generally challenging [37]. We recently described the topological characteristics of hematoxylin and eosin (H&E) images of DLBCL using variable entropy, which is an analysis of the texture of images to measure the randomness statistically. In comparison to reactive lymphoid tissue, DLBCL was characterized by lower entropy. Within the diagnostic category of DLBCL, high entropy was characterized by lower overall survival and death within the first 2 years [37].

Skeletonization is another type of topological analysis that reduces all objects to lines in a 2-D binary image or 3-D binary volume [38,39,40,41]. In the case of 2D images, it reduces all objects to 1-pixel-wide curved lines, without changing the essential structure of the image. This process, called skeletonization, extracts the centerline while preserving the topology [42,43] and Euler number (also known as the Euler characteristic) of the objects [44,45,46]. Skeletonization is a computer vision process that reduces a binary image to its topological skeleton, preserving the image’s shape connectivity and geometry. Skeletonization is widely used for applications such as image description, segmentation, and pattern recognition [47]. To date, skeletonization analysis of histological images of DLBCL has not been performed.

This study analyzed the topological characteristics of DLBCL using hematoxylin and eosin slides in a series of 153 patients, including 110 patients with DLBCL and 43 patients with reactive lymphoid tissue. The study found that DLBCL was characterized by lower skeletonization than reactive lymphoid tissue. In DLBCL, high skeletonization is associated with poor prognosis and enrichment of immuno-oncology markers.

## 2. Materials and Methods

### 2.1. Patients and Samples

A series of 153 patients was selected from the Department of Pathology, Tokai University, School of Medicine. The series included 110 cases of diffuse large B-cell lymphoma and 43 cases of reactive lymphoid tissue and reactive tonsils. The cases were diagnosed according to the current lymphoma classification [3,48,49,50,51,52]. The diagnosis was based on the clinicopathological characteristics of the patients, including the evaluation of the hematoxylin and eosin (H&E) stainings, immunophenotype, and molecular pathology data when required [50,51]. All DLBCL cases were diagnostic biopsies collected from 2007 to 2011. The selection criterion was samples with enough tissue to create a tissue microarray of 6 mm cores. Reactive lymphoid tissue was whole-tissue samples.

This study was conducted according to the guidelines of the Declaration of Helsinki and the World Medical Association for medical experimentation version 24. This study was approved by the Tokai University ethical committee (Institutional Review Board, IRB20/156).

Clinicopathological correlations were performed using the skeletonization variable.

### 2.2. Skeletonization and Area Measurement

Skeletonization reduces binary objects to 1 pixel-wide representations. The skeletonization technique is useful for feature extraction and represents an object (image)’s topology [44,45].

DLBCL images were transformed from hematoxylin and eosin (H&E) into a skeletonized image. H&E had previously been stained using an automated slide stainer (Tissue-Tek Prisma Plus, Sakura Finetek Japan Co., Ltd., Tokyo, Japan) as previously described [53]. The method is thoroughly described from paraffin block to H&E and digitalization in our recent publication [53].

H&E images were digitalized using a digital slide scanner and whole slide imaging system (NanoZoomer S360, #C13220-01, Hamamatsu Photonics K.K., Hamamatsu, Japan) and visualized using NDP.view2 image viewing software (#U12388-01, Hamamatsu Photonics K.K.). From each case, digitized JPEG images were exported at 200× magnification and 150 dpi. For better image processing and performance, each image was split into image patches of 224 × 224 × 3 resolution using PhotoScape v3.7 (http://photoscape.org/; last accessed on 20 June 2026).

All image patches were revised manually by medical pathologist specialist (J.C.), and noninformative patches were excluded. The exclusion criteria were as follows: image patches of size different from 224 × 224 × 3; less than 80% of tissue present; presence of broken, smashed, or other artifacts; and non-diagnostic areas (necrosis and normal lymphoid tissue in lymphoma samples).

The skeletonization process extracted the centerline of an object while preserving the topology and Euler number (also known as the Euler characteristic). The process was initiated on grayscale image patches. The object of interest was dark threads against a light background. Skeletonization required a binary image with white foreground (1) and black background (0) pixels. The complement of the original grayscale image was used. Then, the image was binarized, and skeletonization was performed. In this study, pruning was not performed. Finally, the area of the skeleton was measured for each image patch.

When required only for visualization, the skeleton was overlaid on the original image.

### 2.3. Computer Code

Computer vision analysis was performed using MATLAB (R2026a Update 4 (26.1.0.3312084), 30 June 2026). This study used the ‘bwskel’ function. Morphological operations on binary images using ‘bwmorph’ were also tested, such as the ‘skel’ function that created comparable skeleton images as the ‘bwskel’ function. Although both skel and bwmorph skeletonize 2-D images, they use different algorithms. The ‘bwskel’ function uses 4-connectivity, while ‘bwmorph’ uses 8-connectivity. ‘Bwskel” assumes that foreground objects in the binary image are logically true (white). Therefore, if the original image has a white background and black objects, the complement of the image is used. In this study, we only used the ‘bwskel’ function for the final analysis. The skeletonization codes for one image and a folder with multiple images are shown in Table 1. In this study, small spurs were not pruned. Notably, the ‘bwskel’ function improved the skeletonization algorithm for 2-D images in R2026a: the function omits a padding operation and skeletonizes 2-D images directly without conversion in the third dimension. This new algorithm produces more symmetric and realistic skeletons for 2D images.

### 2.4. Immunohistochemistry

Immunophenotype was performed using conventional immunohistochemical techniques using a Leica Bond Max II automated immunohistochemistry stainer following the manufacturer’s instructions (Leica Biosystems K.K., Tokyo, Japan). Immunohistochemistry reagents included Bond Dewax solution (#AR0084), Bond epitope retrieval solution 2 (#AR0087), primary antibody diluent (#AR9352), and the Bond polymer refine detection kit (#DS9800). Slides were mounted using a fully automated glass coverslipper (Leica CV5030, Leica Biosystems).

All primary antibodies were purchased from Novocastra (Leica Biosystems), including CD20 (clone L26, #CD20-L26-L-CE), CD79a (clone JCB117, #CD79A-599-L-CE), CD3 (clone LN10, #CD3-565-L-CE), CD5 (clone 4C7, #CD5-4C7-L-CE), BCL2 (#BCL-2-L-CE), Ki67 (clone K2, ACK02), and MYC (anti-c-Myc antibody [Y69], ab32072, Abcam K.K., Tokyo, Japan).

The cell of origin characterization using Hans’ algorithm [12] used the combination of CD10 (clone 56C6, #CD10-270-L-CE), BCL6 (clone LN22, # BCL-6-564-L), and MUM1 (clone EAU32, #MUM1-L); more than 30% positivity by neoplastic B lymphocytes was considered positive for calculating Hans’ algorithm.

Immune microenvironment images were retrieved from our previous publications [37,54,55,56], and immunohistochemistry was reanalyzed [37]. The primary antibodies that targeted the immune microenvironment, immune checkpoint and cell cycle were the following [37]: Ki67 (Cell proliferation, clone MM1, Novocastra, Leica), IL10 (Immuno-oncology, immune suppressor, #LS-B7432, Lifespan Bioscience, Seattle, WA, USA), PD-L1 (CD274) (Immuno-oncology, clone E1J2, Cell Signaling, Tokyo, Japan), CSF1R (Immuno-oncology, clone FER216, Spanish National Cancer Research Center (CNIO), Madrid, Spain [56], CD163 (Tumor-associated macrophages, clone 10D6, Novocastra, Leica), CASP8 (Active subunit p18, clone 11B6, Novocastra) [54], TNFAIP8 (Apoptosis, #14559-MM0, Sino Biological, Kawasaki, Kanagawa, Japan) [55], LMO2 (Hematopoietic development, clone 299B, CNIO), MYC (Proto-oncogene, clone Y69, Abcam, Tokyo, Japan), MDM2 (Proto-oncogene, IF2, Invitrogen, Tokyo, Japan), A-B-C MYB (hematopoiesis, DANI51B, CNIO), CDK6 (Cell cycle, clone 98D, CNIO), E2F1 (Cell cycle, clone Agro368V, CNIO), and TP53 (Cell regulation, DO-7, Novocastra, Novocastra). ISY1 primary antibody was purchased from Novus Biologicals (Rabbit Polyclonal, #NBP1-81864). This study is a companion to our recently published entropy, deep learning, and DLBCL analyses [37,57].

Staining was initially evaluated using an ordinal variable as 0, +1, +2, and +3 based on the density of cells positive for each marker. Digital image quantification using Fiji software was undertaken to assess the total percentage of positive cells (ImageJ 1.54p, Wayne Rasband and contributors. National Institutes of Health, USA. Java 1.8.0_322 (64-bit)). In summary, a large representative area was digitized, and the number of DAB-positive pixels was identified in the blue stack. The percentage of positive cells was calculated as follows: percentage = ([positive pixels/all pixels] × 100) [58].

The function of all markers is listed in Table 2.

### 2.5. In Situ Hybridization

Characterization of the gene rearrangement and translocation status was performed using DNA fluorescent in situ hybridization (FISH) break-apart probes targeting *MYC*, *BCL2*, and *BCL6* genes (Vysis, Abbott Japan Co., Ltd., Tokyo, Japan).

RNA in situ hybridization was used to detect Epstein–Barr virus (EBV) infection using Bond in situ hybridization probes targeting EBV-encoded RNA (EBER) (#PB0589, Leica Biosystems, Tokyo, Japan).

### 2.6. Gene Expression

RNA was extracted from formalin-fixed paraffin-embedded (FFPE) tissue sections of 10 micrometer thickness using the RNeasy FFPE Kit (50) (#73504, Qiagen, Tokyo, Japan). Before RNA extraction, histology was revised, and cases with at least 80% of neoplastic areas were selected. After quality control, the RNA was used to quantify gene expression levels using the nCounter^®^ PanCancer Profiling Panel (Bruker Spatial Biology, Bruker Corporation, Billerica, MA, USA). The tissue sections were outsourced to Celgene Corporation (Summit, NJ, USA) for this analysis. This panel included 730 immune-oncology genes and 40 housekeeping genes.

The analysis was standard, including the calculation of log2 fold changes and −log10 *p*-values to create a volcano plot and, later, a heatmap, as well as hierarchical clustering using the one-minus Pearson correlation and average linkage method.

During the analysis of raw data, calibrated data were normalized to the positive control. Calibrated data resulted from multiplying raw data by calibration factors. Housekeeping gene normalization was performed as follows: calculate the geometric mean of the housekeeping genes for each lane (or each sample), divide counts for each gene by the lane (or sample) specific geometric mean, and convert to the log2 scale (log2((normData[,i]/hkGeoMeans[i]))) or log2((normData[,i]/hkGeoMeans[i] × scaling factor)), where the scaling factor could be a constant such as 1000.

### 2.7. Functional Network Association Analysis

STRING version 12.0 was used to create a protein–protein functional network association analysis. Functional enrichment used the biological process (Gene Ontology) category; similarity ≥ 0.8 was used for grouping terms, and terms were sorted by signal. K-means clustering with 4 clusters was used to find the defined number of clusters based on their centroids. Confidence strength was shown as the thickness of the network edges. The minimum required interaction score was set at 0.4.

### 2.8. Gene Set Enrichment Analysis

Gene set enrichment analysis (GSEA) was performed using GSEA 4.4.0 software (build 18) (Broad Institute and UC San Diego, San Diego, CA, USA) [119,120,121]. GSEA used 3 types of files: phenotype.cls, gene expression gct, and gene sets gmx. The parameters were standard, including 1000 permutations, collapse to gene symbols, phenotype permutation type, weighted enrichment statistics, signal-to-noise metric for ranking genes, real gene list sorting mode, descending gene list ordering mode, 1000 maximum size of sets, and 10 minimum size of the smaller set. The NanoString chip file was manually created. Hallmark gene sets were retrieved from the Molecular Signatures Database (MSigDB). All methodology was performed as previously described [122,123].

### 2.9. Statistical Analysis

Comparison between groups used nonparametric tests, including the Mann–Whitney U test for 2-independent samples test and Kruskal–Wallis H test for several (≥3) independent samples. Crosstabulation was used to correlate qualitative variables with the Pearson chi-square test and Fisher’s exact test. Overall survival was calculated from the time of diagnosis and biopsy to the date of death or last follow-up using the Kaplan–Meier and log-rank tests. A *p*-value of less than 0.05 was considered statistically significant.

All statistical analyses were performed using IBM SPSS Statistics version 27 (Release 27.0.1.0, 64-bit edition; 1 Orchard Road, Armonk, NY, USA).

## 3. Results

### 3.1. Clinicopathological Characteristics

This series corresponds to a conventional series of large B-cell lymphoma (DLBCL). The clinicopathological characteristics of the series were as follows: age > 60, 77/110 (70%); male, 57/110 (51.8%); Hans’ classification cell of origin non-GCB, 75/108 (69.4%); CD5-positive, 13/109 (11.9%); Epstein–Barr virus EBER positive, 28/108 (25.9%); nodal (+spleen), 57/110 (51.8%); International Prognostic Index (IPI) high + high/intermediate, 29/87 (33.3%); high sIL2R, 76/96 (79.2%); and RCHOP + RCHOP-like treatment, 89/94 (94.7%).

By FISH, *MYC* rearrangement was found in 5/94 (5.3%) and *BCL2* rearrangement in 3/93 (3.2%). No double-hit cases were included in the series (Table 3).

### 3.2. Evaluation of Skeletonization in Test Images

**Figure 1:**
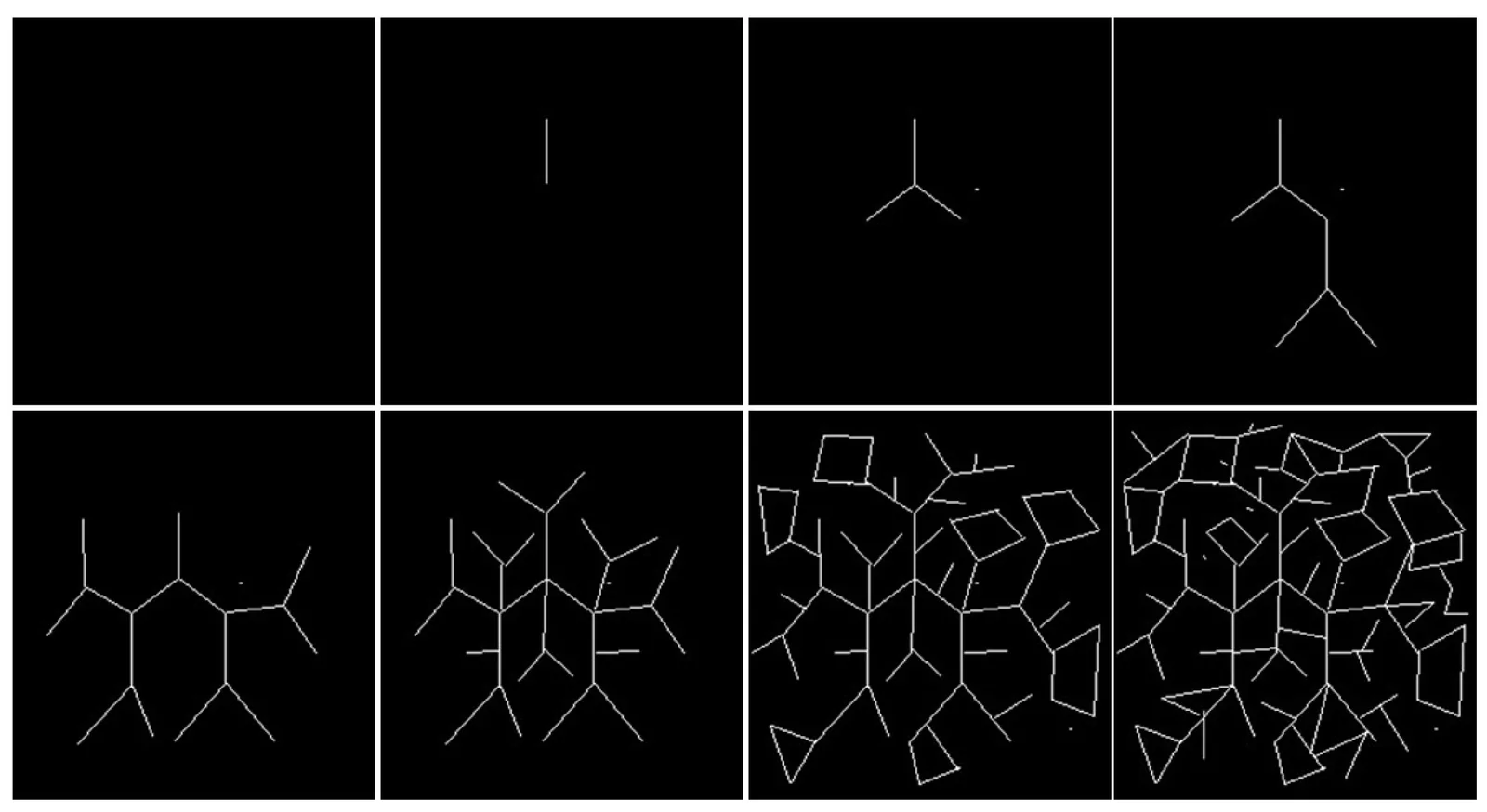
Skeletonization on test images. Skeletonization can be used for feature extraction and represents an object’s topology. Skeletonization area measurement was tested on a set of 8 images to evaluate the usefulness of the ‘bwskel’ function. The area values from images 1–8 were as follows: 0, 40, 100, 215, 526, 857, 1786, and 2603, respectively. Therefore, the function measured skeletonization complexity in a correct manner.

Skeletonization reduces binary objects to 1 pixel-wide representations. The skeletonization technique is useful for feature extraction and represents an object (image)’s topology. Skeletonization area measurement was tested on a set of eight images to evaluate the usefulness of the ‘bwskel’ function in conceptual conditions (i.e., from low to high skeletonization complexity). The area values from images 1–8 were as follows: 0, 40, 100, 215, 526, 857, 1786, and 2603, respectively. Therefore, the function measurement was conceptually correct (Figure 1).

**Figure 2:**
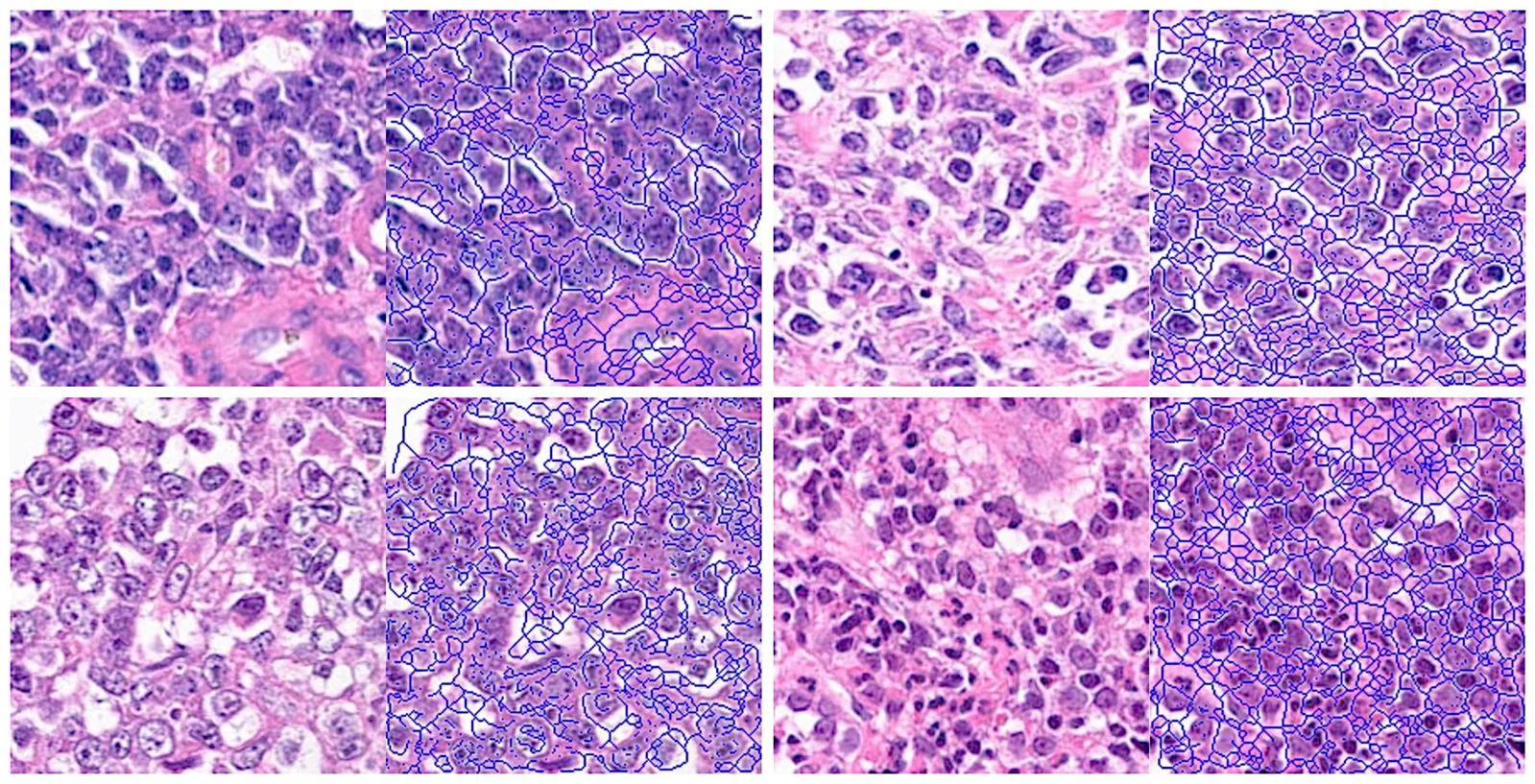
Skeletonization on test images of DLBCL. Skeletonization can be used for feature extraction and represents an object’s topology. Skeletonization is a computer vision process that reduces a binary image to its topological skeleton, preserving the image’s shape connectivity and geometry. Skeletonization is widely used for image description, segmentation, and pattern recognition. In the case of DLBCL, high histological skeletonization is a measure of heterogeneity. The curves delineate the borders of the cells at the cytoplasmic-membrane cellular structure. In addition, immune microenvironmental cells such as lymphocytes and neutrophils are delineated as well as necrotic cells, cells undergoing apoptosis, and other cellular components in the extracellular matrix. In summary, large homogeneous neoplastic B lymphocytes with a less complex microenvironment result in lower skeletonization. Morphologically heterogeneous neoplastic B lymphocytes with a more complex microenvironment and extracellular matrix result in higher skeletonization. Original magnification 200×.

Examples of the skeletonization of DLBCL images are shown in Figure 2.

### 3.3. Evaluation of the Skeletonization Area in DLBCL

#### 3.3.1. Difference Between DLBCL and Reactive Lymphoid Tissue

**Figure 3:**
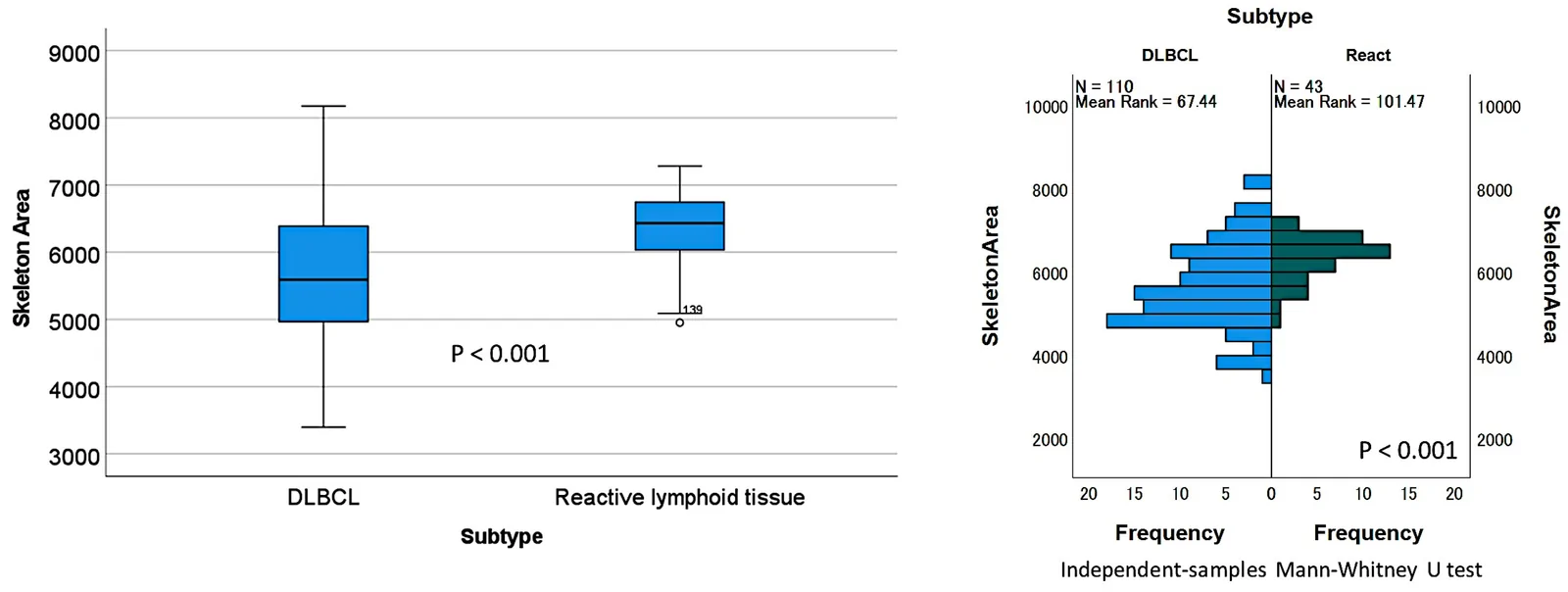
Different skeletonization areas between DLBCL and reactive lymphoid tissue. Skeletonization technique is useful for feature extraction and representation of an object’s topology. In comparison to reactive lymphoid tissue, DLBCL was characterized by a lower skeletonization area, 5665.20 ± 1012.82 vs. 6341.16 ± 548.23 respectively (*p* < 0.001).

The skeletonization technique is useful for feature extraction and represents an object’s topology. The DLBCL skeleton area ranged from 3396.12 to 8176.94, with a mean of 5665.20 ± 1012.82 and a median of 5591.65. In comparison to reactive lymphoid tissue, DLBCL was characterized by a lower skeletonization area, 5665.20 ± 1012.82 vs. 6341.16 ± 548.23 respectively (*p* < 0.001) (Figure 3).

#### 3.3.2. Correlation Between Skeletonization and Overall Survival of the DLBCL Patients

**Figure 4:**
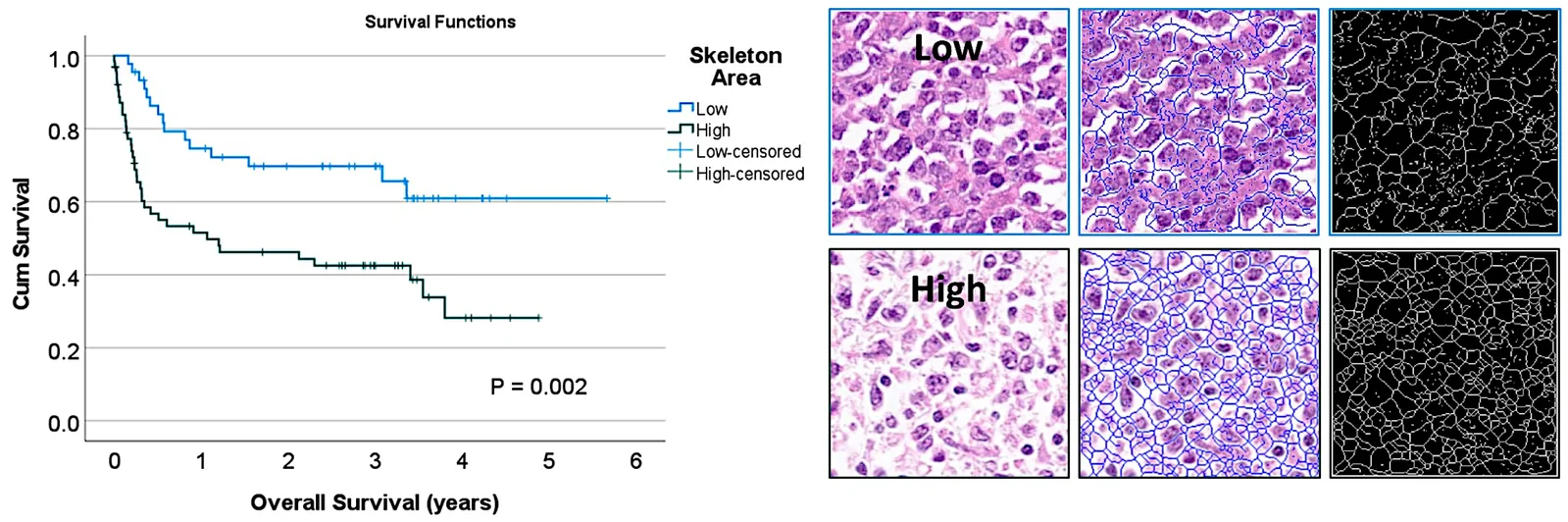
High skeletonization correlated with poor overall survival of DLBCL. Overall survival analysis using the Kaplan–Meier and log-rank tests showed that high skeletonization cases were associated with worse overall survival (*p* = 0.002; Hazard risk = 2.48). Original magnification 200×.

The skeletonization area correlated with the overall survival of patients with DLBCL, and the cases were classified into 2 groups: low skeletonization (45/110, 40.9%) and high skeletonization (65/110, 59.1%). Overall survival analysis using the Kaplan–Meier and log-rank test showed that high skeletonization cases were associated with worse overall survival than cases with low skeletonization (log-rank (Mantel–Cox) *p*-value = 0.002). High skeletonization patients had a hazard risk of 2.484 (95% CI for Exp(B) = 1.362–4.531) (*p* = 0.003) (Figure 4).

**Figure 5:**
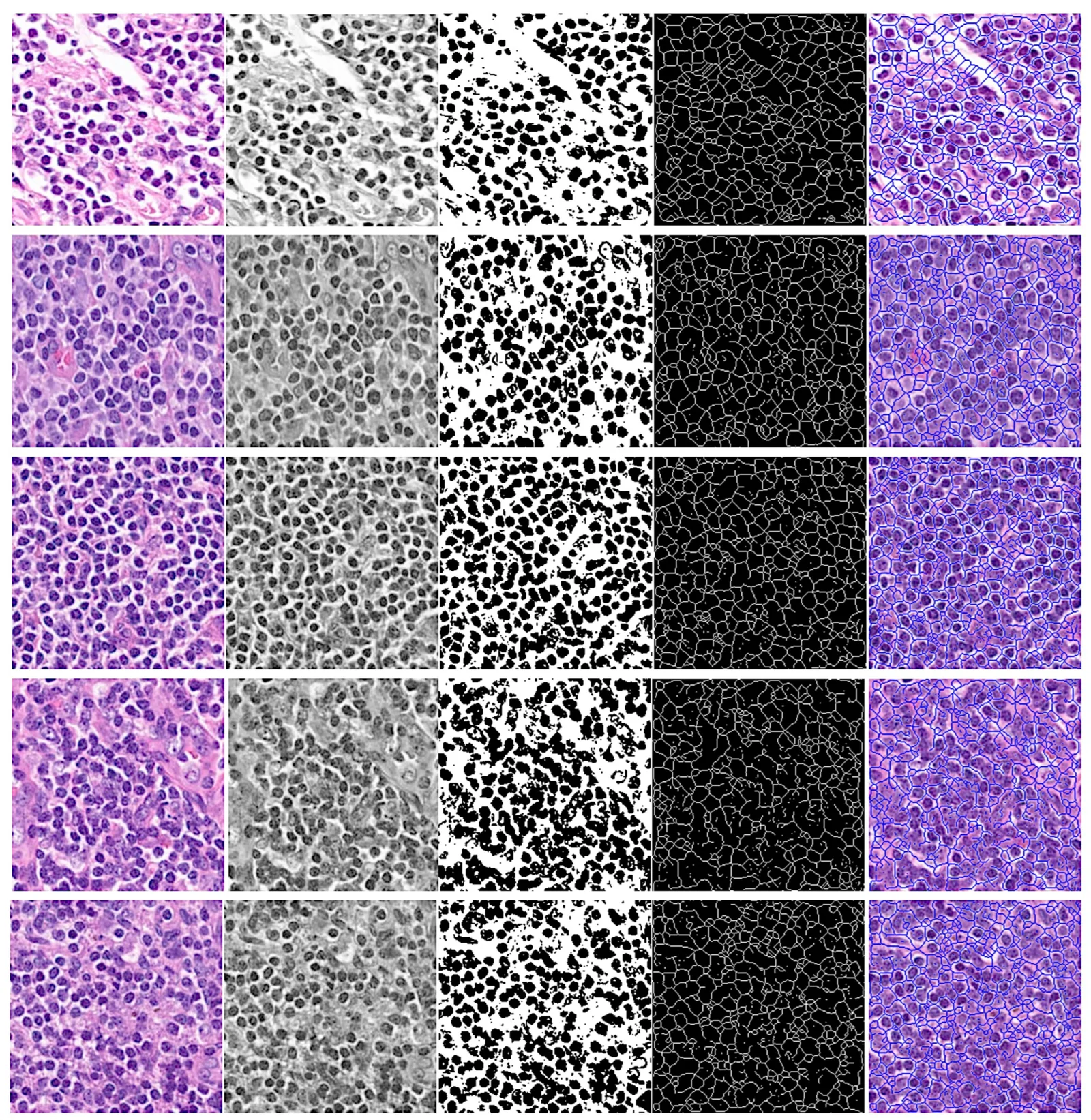
Skeletonization of reactive lymphoid tissue. Skeletonization reduces binary objects to 1-pixel-wide curved line representations without changing the essential structure of the image. This is useful for feature extraction and represents an object’s topology. This study used the skeletonization function to analyze the topological heterogeneity of reactive lymphoid tissue and DLBCL. This figure shows images of reactive lymphoid tissue characterized by higher skeletonization (heterogeneity) than DLBCL. Original magnification 200×.

**Figure 6:**
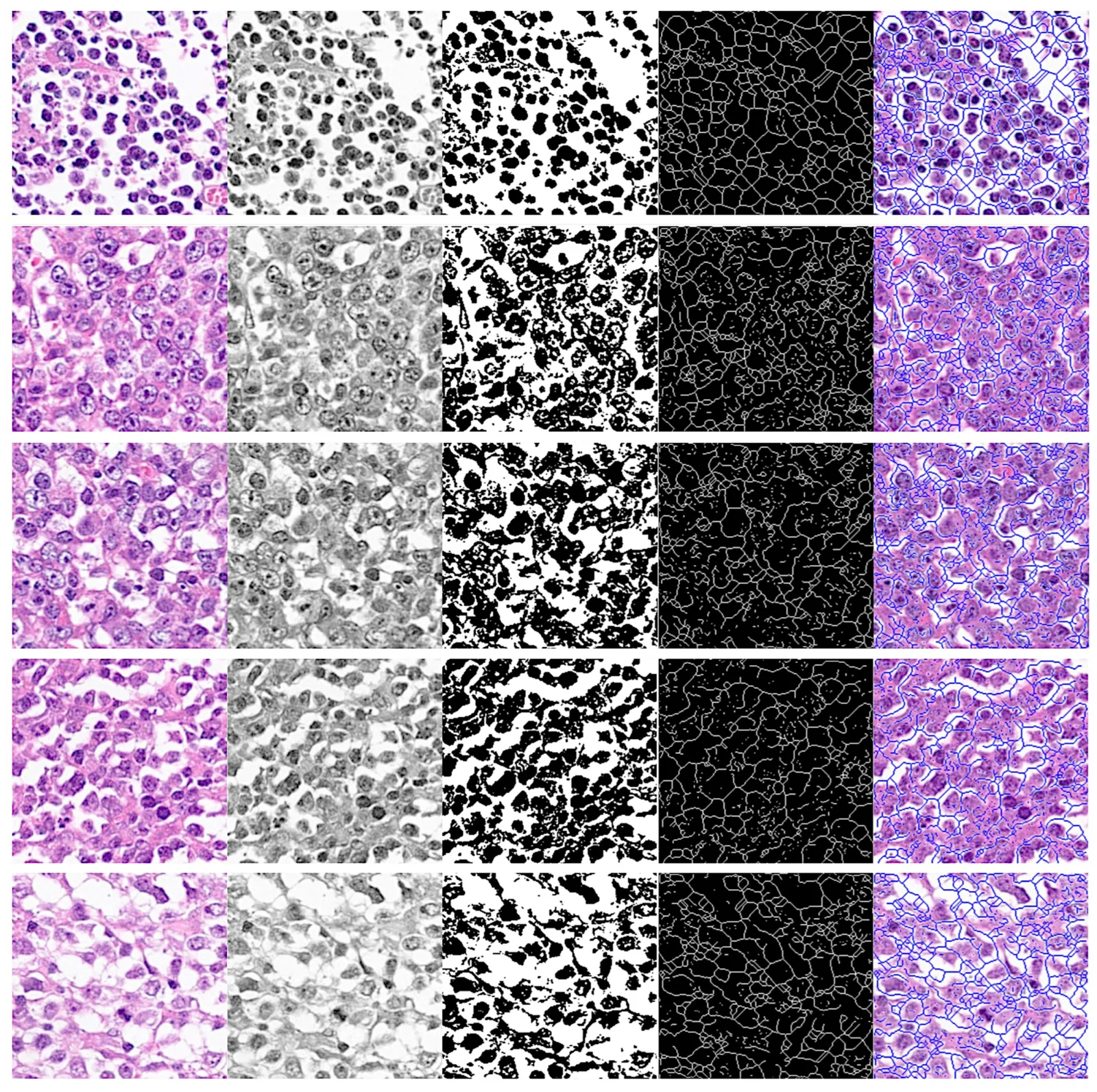
Low skeletonization in the DLBCL. Skeletonization reduces binary objects to 1-pixel-wide curved line representations without changing the essential structure of the image. This is useful for feature extraction and represents an object’s topology. This study used the skeletonization function to analyze the topological heterogeneity of reactive lymphoid tissue and DLBCL. This figure shows images of DLBCL with low skeletonization (heterogeneity). Noteworthy, large homogeneous neoplastic B lymphocytes with a less complex microenvironment result in lower skeletonization. Original magnification 200×.

**Figure 7:**
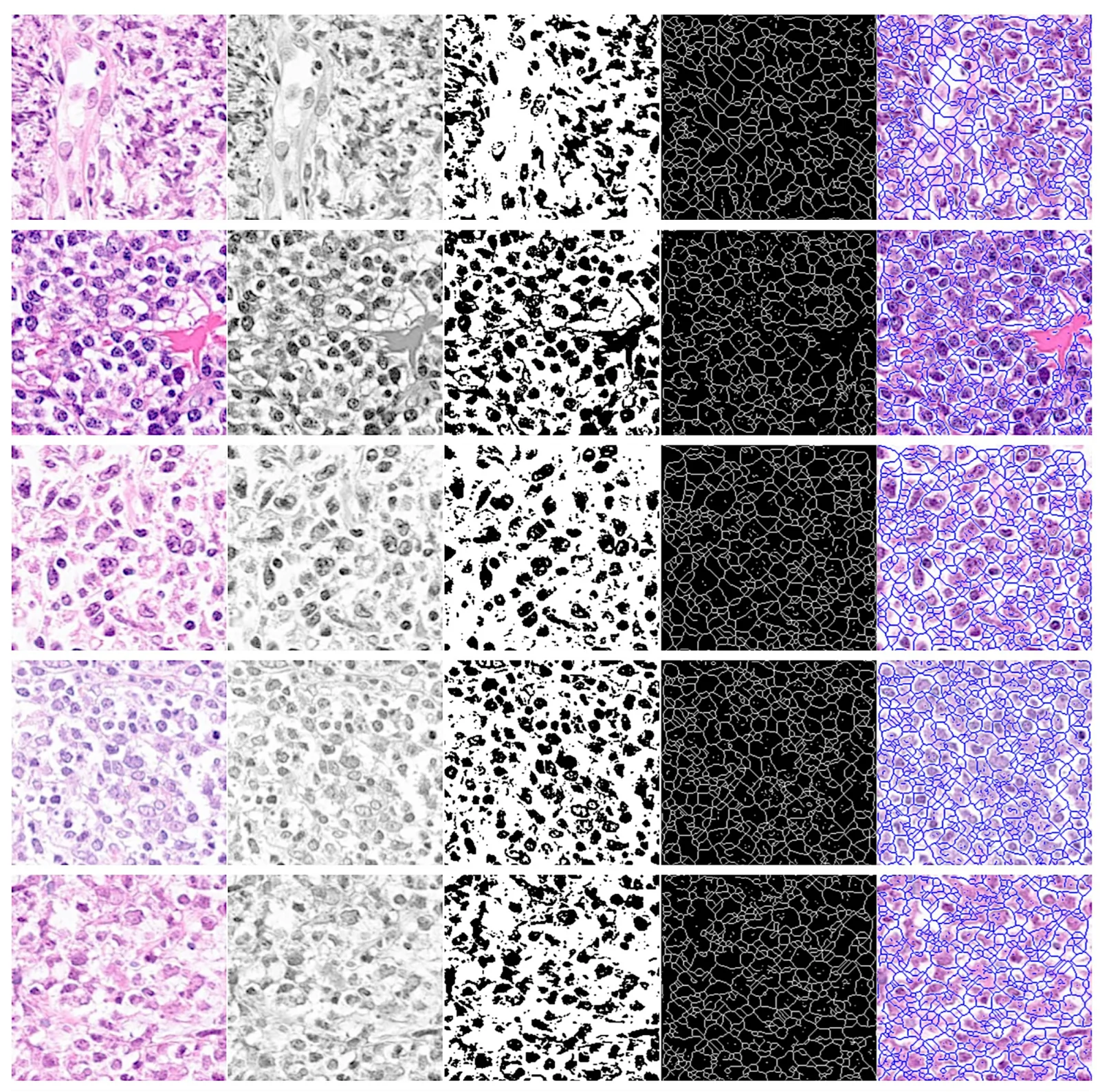
High skeletonization in the DLBCL. Skeletonization reduces binary objects to 1-pixel-wide curved line representations without changing the essential structure of the image. This is useful for feature extraction and represents an object’s topology. This study used the skeletonization function to analyze the topological heterogeneity of reactive lymphoid tissue and DLBCL. This figure shows images of DLBCL with high skeletonization (heterogeneity). Noteworthy, morphologically heterogeneous neoplastic B lymphocytes with a more complex microenvironment and extracellular matrix result in higher skeletonization. Original magnification 200×.

**Figure 8:**
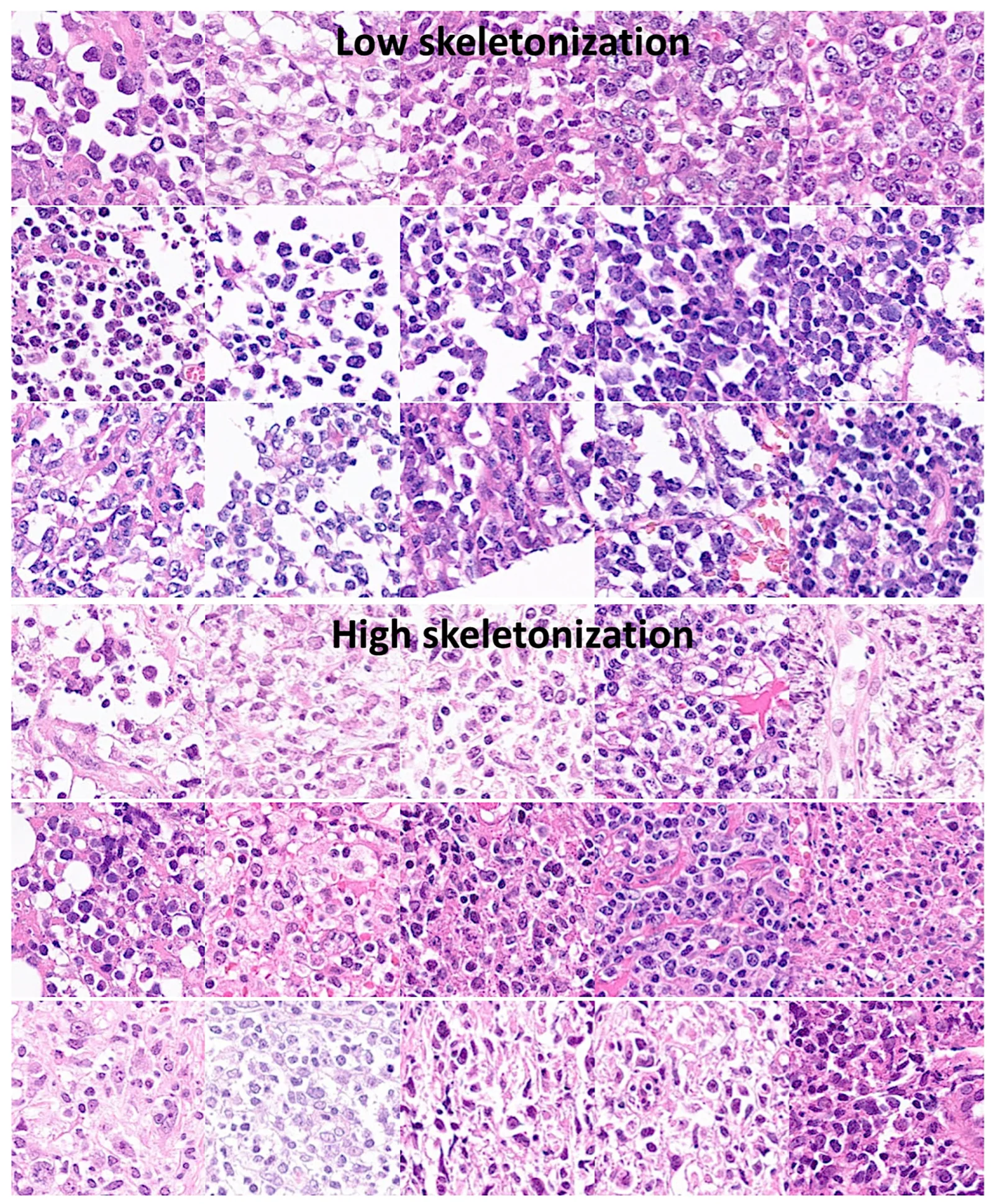
Additional images and differences between low and high skeletonization cases (H&E). In comparison with high skeletonization cases, low skeletonization cases had a DLBCL with more homogeneous and diffuse histological morphology (centrobastic and immunoblastic) and less conspicuous microenvironment and extracellular components during H&E evaluation. Original magnification 200×.

**Figure 9:**
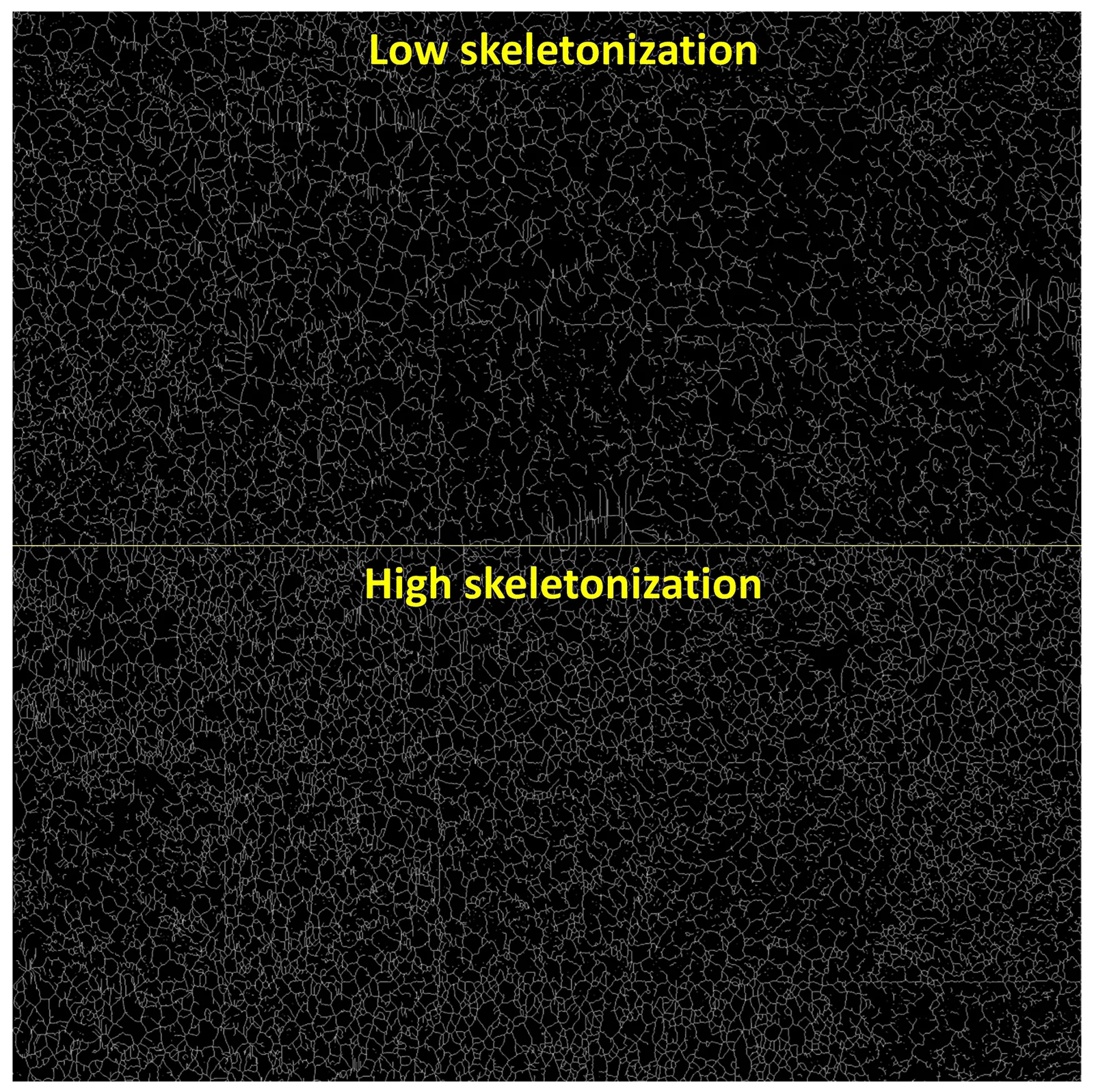
Additional images and differences between low and high skeletonization cases (H&E). High skeletonization cases show a denser filament structure of the skeleton, which correlates with heterogeneous histological cells and topological structure. Original magnification 200×.

Examples of reactive, high, and low skeletonization in DLBCL are shown in Figure 5, Figure 6, Figure 7, Figure 8 and Figure 9.

#### 3.3.3. Correlation Between Skeletonization and Clinicopathological Features of DLBCL Patients

The skeletonization was correlated with several clinical and pathological variables. High skeletonization correlated with higher EBER positivity (*p* < 0.001), lower CD5 positivity (*p* = 0.006), lower E2F1 percentage (*p* < 0.001), lower BCL2 percentage (*p* = 0.010), lower ISY1 percentage (*p* = 0.012), and lower TNFAIP8 (*p* = 0.014). High skeletonization also correlated with a dead event before the 2 years of overall survival follow-up (*p* = 0.014).

A Pearson correlation was run to determine the relationship between skeletonization and entropy in DLBCL. There was a medium, positive correlation between skeletonization and entropy, which was statistically significant (r = 0.28, n = 109, *p* = 0.003).

Multivariate COX regression analysis for overall survival including IPI, cell of origin Hans’ classifier, EBER, and skeletonization showed that both Hans’ classifier and skeletonization retained prognostic significance.

Multivariate COX analysis including entropy and skeletonization variables confirmed that both variables maintained the prognostic association with overall survival (hazard-risks = 2.44 and 2.45, respectively; all *p*-values < 0.001).

Multivariate COX analysis including EBER and skeletonization confirmed both variables as independent prognostic markers.

Finally, bivariate Pearson correlation between skeletonization and the rest of the markers was performed.

**Figure 10:**
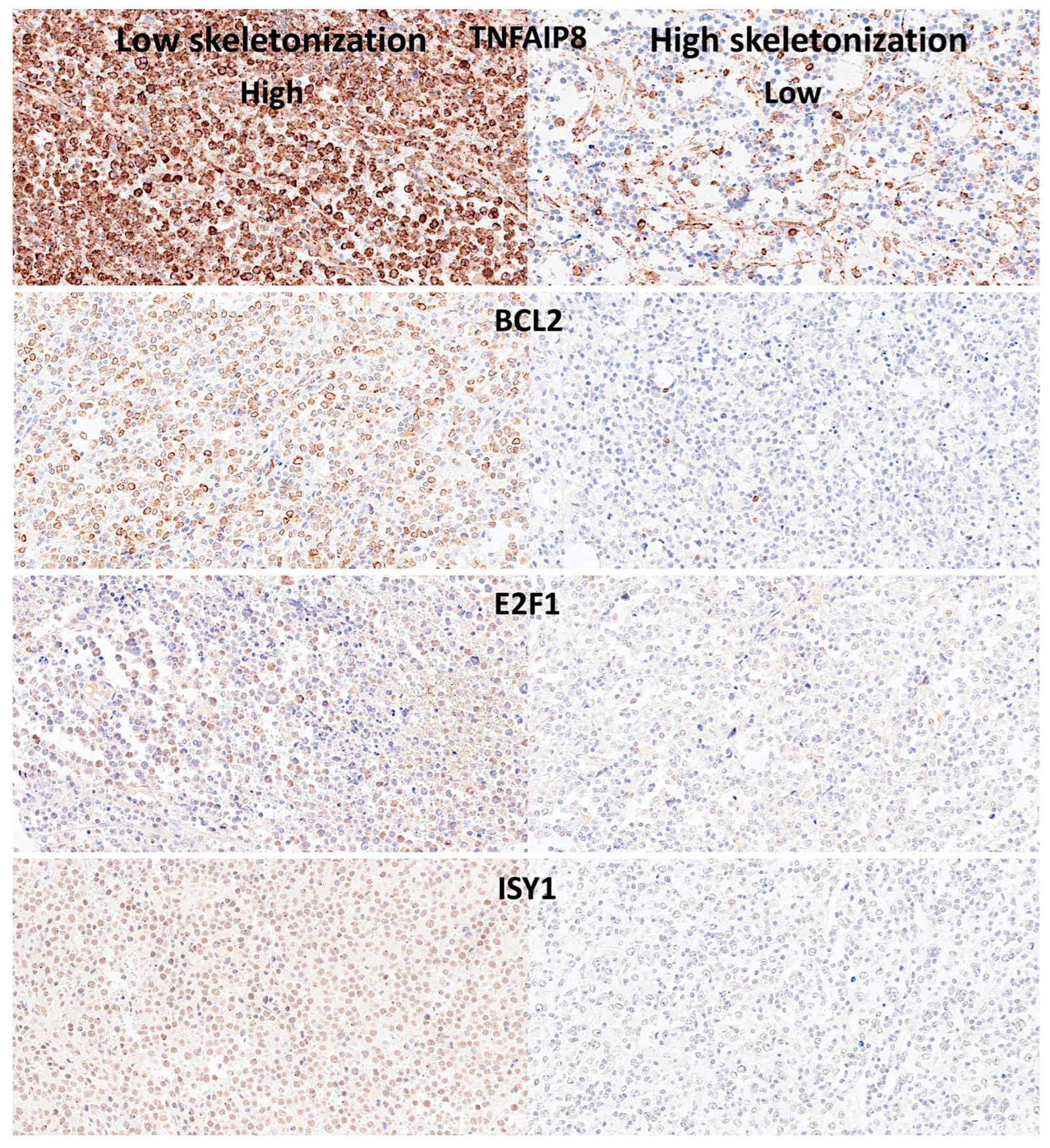
Immunohistochemical images. The skeletonization was correlated with several clinical and pathological variables. High skeletonization correlated with higher EBER positivity (*p* < 0.001), lower CD5 positivity (*p* = 0.006), lower E2F1 percentage (*p* < 0.001), lower BCL2 percentage (*p* = 0.010), lower ISY1 percentage (*p* = 0.012), and lower TNFAIP8 percentage (*p* = 0.014). Original magnification 200×.

**Figure 11:**
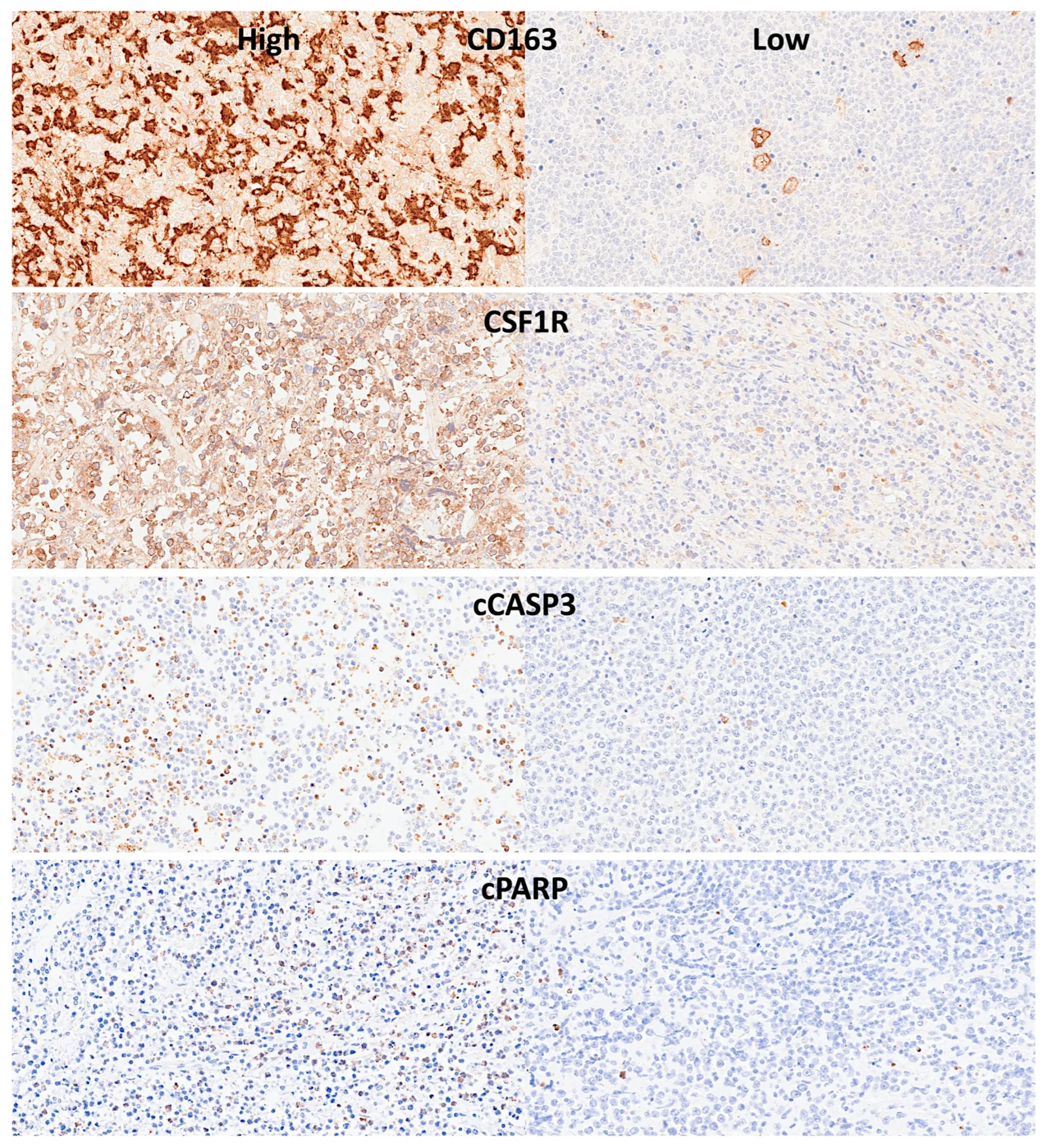
Immunohistochemical images. The skeletonization was correlated with several DLBCL pathological markers, including CD163 (macrophages), CSF1R (macrophages), cCASP3 (apoptosis), and cPARP (apoptosis). Skeletonization did not correlate with these markers. Original magnification 200×.

**Figure 12:**
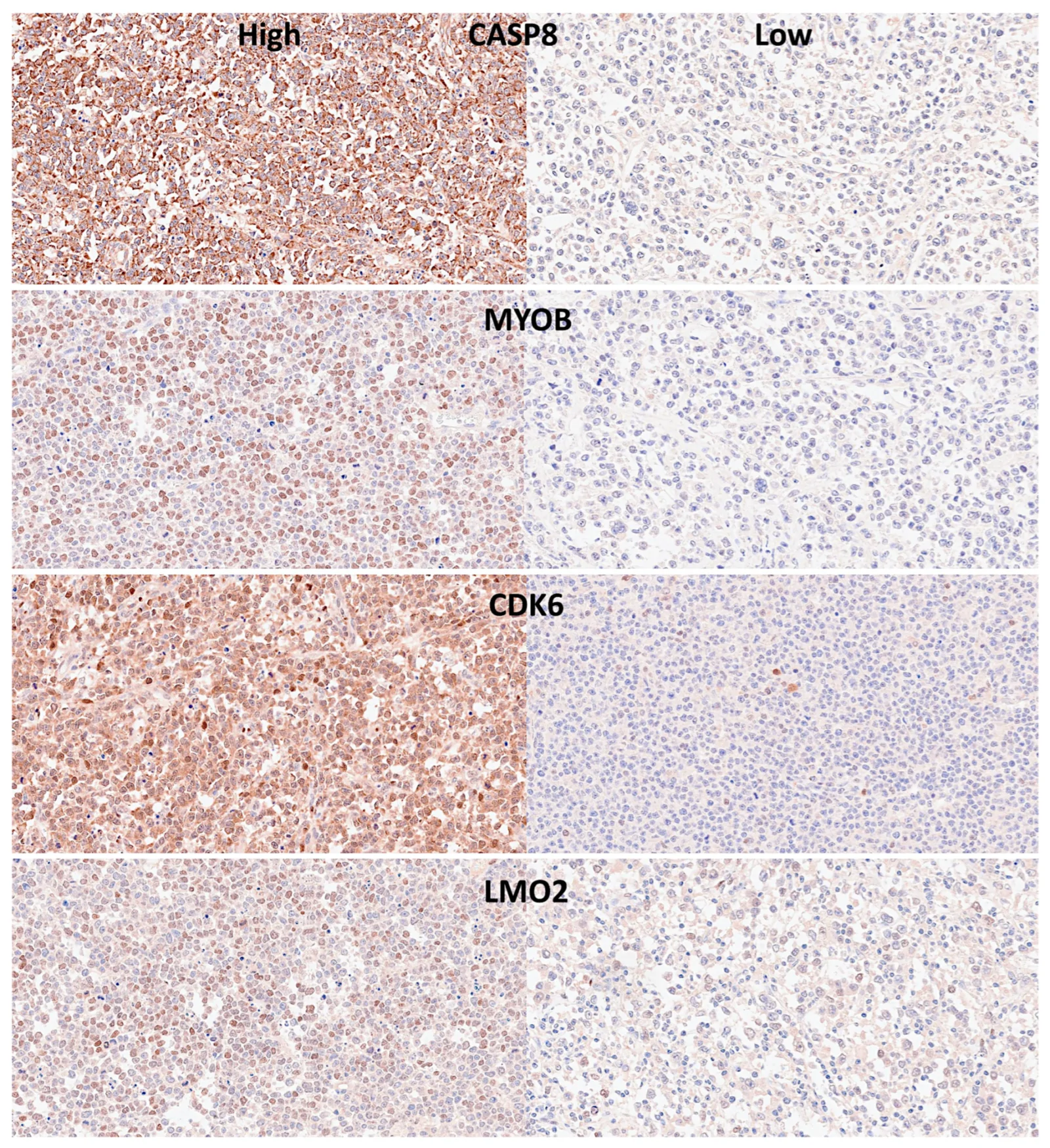
Immunohistochemical images. The skeletonization was correlated with several DLBCL pathological markers, including CASP8 (apoptosis), MYOB (hematopoiesis), CDK6 (cell cycle), and LMO2 (germinal center). Skeletonization did not correlate with these markers. Original magnification 200×.

**Figure 13:**
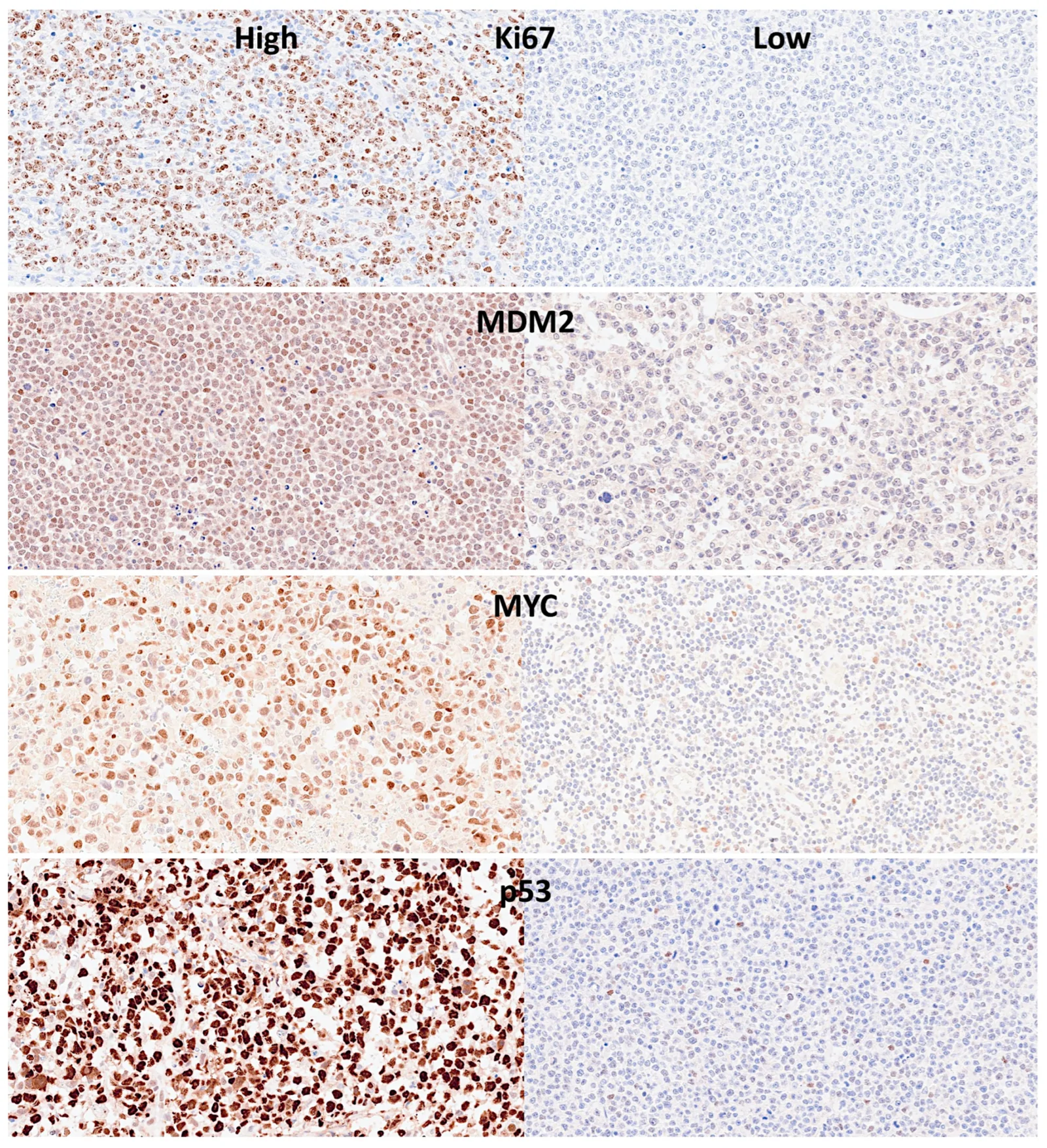
Immunohistochemical images. The skeletonization was correlated with several DLBCL pathological markers, including Ki67 (proliferation), MDM2 (cell cycle), MYC (cell cycle), and p53 (tumor suppressor gene, cell cycle). Skeletonization did not correlate with these markers. Original magnification 200×.

All data are shown in Table 4, Table 5, Table 6, Table 7, Table 8, Table 9, Table 10 and Table 11 and Figure 10, Figure 11, Figure 12 and Figure 13.

#### 3.3.4. Gene Expression Analysis

Gene expression analysis using a pan-cancer immuno-oncology panel of 730 genes was available in a subset of 30 cases, including 18 cases with high skeletonization and 12 cases with low skeletonization. The clinicopathological characteristics are shown in Table 12.

Conventional biostatistics were performed, including the calculation of differentially expressed *p*-values and fold changes. The analysis showed that 166 immuno-oncology genes were upregulated in the high skeletonization group and 8 genes were upregulated in the low skeletonization group.

The first 10 most significant in the high skeletonization group were *C4BPA* (complement), *MUC1* (CD molecules), *IL13RA1* (chemokines, T-cell functions), *RRAD* (cell functions), *CXCL1* (chemokine, regulation), *PLAUR* (CD molecule), *IL22* (anti-inflammatory cytokine), *TLR3* (innate immune response, Toll-like receptor), and *PVR* (regulation of immune response).

**Figure 14:**
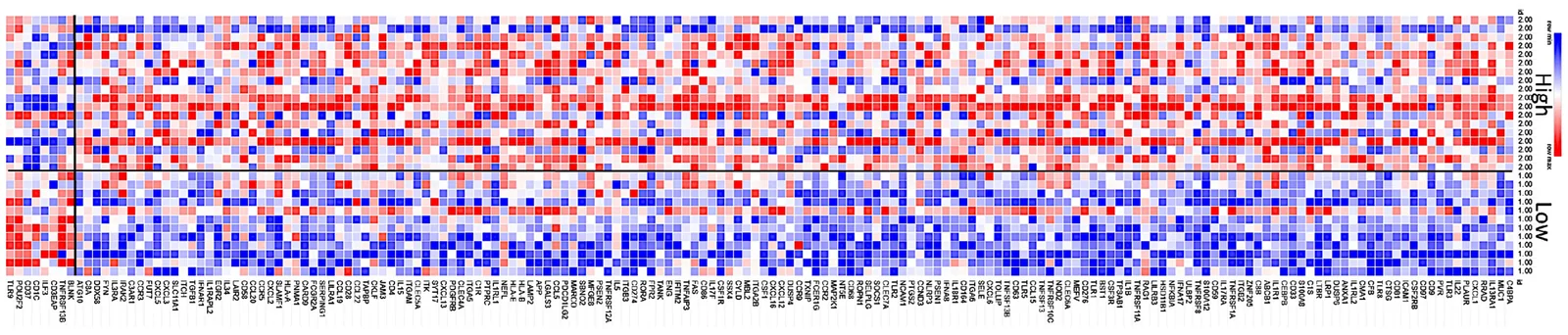
Differential gene expression. This figure shows the heatmap and differential gene expression between high and low skeletonization. The analysis showed that 166 immuno-oncology genes were upregulated in the high skeletonization group and 8 genes were upregulated in the low skeletonization group.

The 8 genes upregulated in the low skeletonization group were *BLNK* (humoral immune response), *TNFRSF13B* (TNF superfamily), *CD3EAP* (adaptive immune response), *ILF3* (chemokine), *CD1C* (T-cell function), *CD37* (adaptive immune response), *POU2F2* (humoral immune response), and *TLR9* (innate immune response, toll-like receptor) (Figure 14).

**Figure 15:**
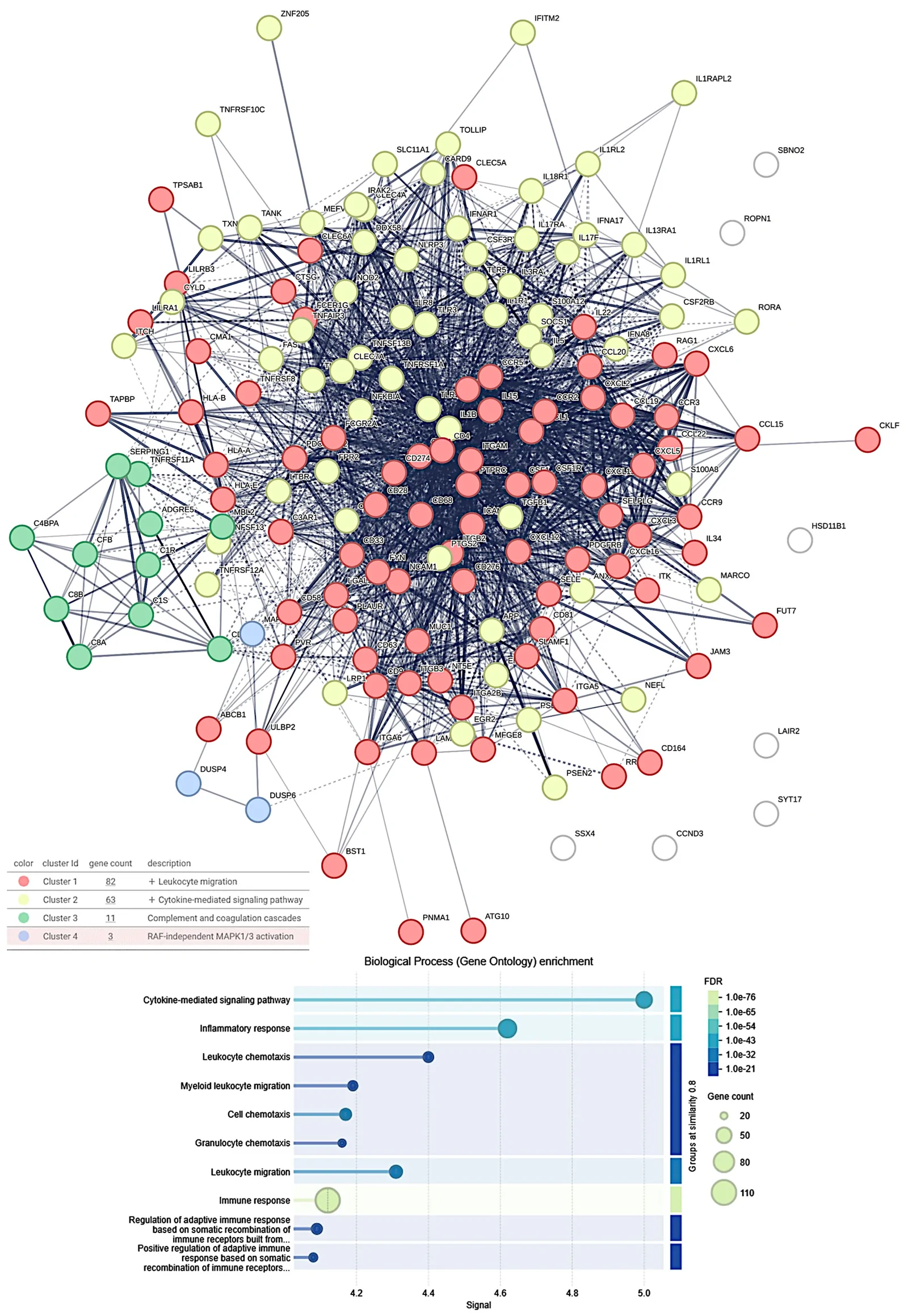
Functional network association analysis of upregulated genes in the high skeletonization group (bad prognosis). Genes located at the center of the hub are potentially more relevant for disease pathogenesis because they have more connections with many genes. Relevant genes with known pathogenesis in DLBCL were *CD274*, *NFKB1A*, *CD68*, *CSF1R*, *CCR5*, *ITGAM* and *TGFB1* (among others).

A functional network association analysis was performed to identify the genes that were more relevant within the high skeletonization group. Within the upregulated genes of the high skeletonization group, relevant genes with known pathogenesis in DLBCL were *CD274* (*PD-L1*), *NFKB1A*, *CD68*, *CSF1R*, *CCR5*, *ITGAM*, and *TGFB1* (among others). The network is shown in Figure 15.

**Figure 16:**
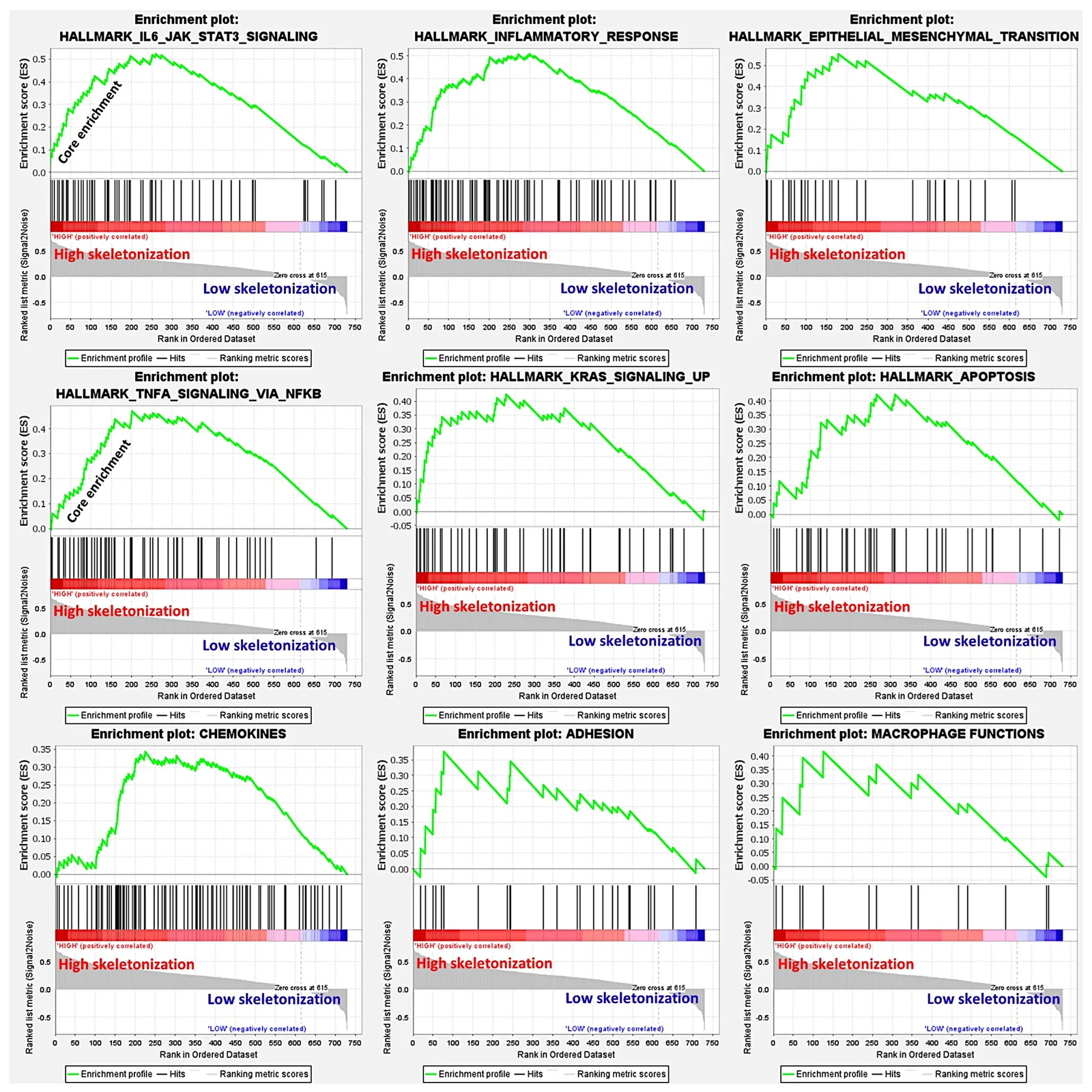
Pathway enrichment in the high skeletonization group. By gene set enrichment analysis (GSEA), there was enrichment of different pathways in the high skeletonization group, including IL6 JAK STAT3 signaling, inflammatory response, TNF, NFKB, KRAS, apoptosis, and macrophage function.

By GSEA, there was enrichment of different pathways in high skeletonization, including IL6 JAK STAT3 signaling, inflammatory response, TNF, NFKB, KRAS, apoptosis, and macrophage function (Figure 16).

## 4. Discussion

DLBCL is the most common histological subtype of non-Hodgkin lymphoma (NHL) and accounts for approximately 35 of NHL [2,3,9]. The molecular pathogenesis is complex and a multistep process initially initiated by the neoplastic transformation of germinal center or post-germinal B lymphocytes. Most of the steps of neoplastic transformation are still unknown. One of the best-known oncogenic events is the rearrangement of *MYC*, *BCL2*, and *BCL6* genes. This study included the study of rearrangements in most of the cases. However, the rearrangement status did not correlate with skeletonization.

The diagnostic category of DLBCL was previously used to describe a group of lymphomas with heterogeneous morphology, genetics, and biological behavior. There are several distinct large B-cell lymphoma variants, including primary mediastinal large B-cell lymphoma (PMBL), T-cell-rich large B-cell lymphoma, intravascular large B-cell lymphoma, lymphomatoid granulomatosis, Epstein–Barr virus (EBV) positive DLBCL, DLBCL associated with chronic inflammation, fibrin-associated large B-cell lymphoma, primary large B-cell lymphoma of immune-privileged sites (central nervous system, vitreoretinal, and testis), fluid overload-associated large B-cell lymphoma, DLBCL associated with IRF4 rearrangements, and ALK-positive large B-cell lymphoma.

This study of 110 cases of DLBCL did not differentiate between all these subtypes but used the DLBCL “not otherwise specified” (NOS) diagnostic category, and the DLBCL cases were classified according to their primary site as nodal (plus spleen), Waldeyer’s ring, gastrointestinal, and other extranodal. When a correlation was made with the skeletonization level, no differences were found. However, skeletonization correlated with the presence of Epstein–Barr virus (EBV): low, 4/44 (9.1%) vs. high, 24/64 (37.5%) (*p* < 0.001). Therefore, our data indicate that in a subset of cases with virus infection, the level of skeletonization (understood as heterogeneity as well) depends on pathogenic mechanisms associated with EBV. Notwithstanding, 72.5% of high skeletonization cases were not associated with EBV. Therefore, other mechanisms may be associated.

Our series is enriched with Epstein–Barr virus (EBER) positive cases, and the association of EBER with skeletonization may create a bias. To check this situation, a multivariate COX regression analysis for overall survival was performed, including skeletonization and EBER, and the results confirmed that both variables were statistically significant. Therefore, the prognostic value of skeletonization is independent of EBER.

Gene expression has been extensively studied in DLBCL and led to the identification of three main types: germinal center B-cell-like (GCB), activated B-cell-like (ABC), and not-otherwise-specified type 3 [124,125,126]. In this study, we used the surrogate immunohistochemical-based cell-of-origin classification of Hans that differentiates two groups, GCB and non-GCB. The non-GCB DLBCL is associated with poor prognosis [12,13,127]. We speculated that the high skeletonization group associated with poor prognosis would also correlate with the non-GCB subtype. Nevertheless, that was not the case. Other factors may be associated with the histological subtype.

We correlated skeletonization level with other markers of the immune microenvironment and cell cycle and found that high skeletonization also correlated with lower CD5, E2F1, BCL2, ISY1, and TNFAIP8 protein levels (all *p* values < 0.05). We previously described that high immunohistochemical expression of TNFAIP8 is associated with poor prognosis in DLBCL [55]. To deepen the pathological background of high skeletonization and its association with the prognosis of DLBCL patients, we performed gene expression analysis using a pan-cancer immune profiling panel. We found that 166 immuno-oncology genes were upregulated in the high group and only 8 genes were upregulated in the low skeletonization group. Therefore, the high skeletonization enriches immuno-oncology markers. This association is important as immunotherapy in DLBCL has gained interest recently [128,129,130,131].

A functional network association analysis was performed to identify the genes that were more relevant within the high skeletonization group. Within the upregulated genes of the high skeletonization group, relevant genes with known pathogenesis in DLBCL were *CD274* (*PD-L1*), *NFKB1A*, *CD68*, *CSF1R*, *CCR5*, *ITGAM*, and *TGFB1* (among others). We have previously described the relevance of these genes in DLBCL. For instance, high PD-L1 levels were associated with poor overall survival of DLBCL [55], high PTX3 and CD68-positive macrophages were correlated with poor survival of DLBCL [58], CSF1R correlated with the prognosis of the DLBCL patients in a bimodal manner [56], and immune-oncology markers were associated with aggressive DLBCL [28,29].

Revisiting the morphology of DLBCL has regained interest recently [26,132]. This study analyzed DLBCL from a different approach using skeleton analysis. Further development of the technique is warranted.

## 5. Limitations

This study used the skeletonization function to analyze the topological heterogeneity of DLBCL in a series of 153 patients, including 110 patients with DLBCL and 43 patients with reactive lymphoid tissue. This study has the limitation of the number of cases. A larger series of cases with gene expression data will be needed to confirm our findings.

## 6. Conclusions

DLBCL is characterized by lower skeletonization than reactive lymphoid tissue. In DLBCL, high skeletonization is associated with poor prognosis and enrichment of immuno-oncology markers.

## Figures and Tables

**Table 1:** Skeletonization code.

%Code for One Image	%Code for Folder
clc clear I = imread(“***”); imageshow(I) G = rgb2gray(I) imageshow(G) BW = imbinarize(G) imageshow(BW) B = bwskel(BW); stats = regionprops(B, “Area”); areaValue = sum([stats.Area])	imageLoc = “skeletontraining”; ds = imageDatastore(imageLoc); for i = 1:length(ds.Files) I = readimage(ds, i); G = rgb2gray(I); B = imbinarize(G); S1 = bwskel(B); imageshow(S1) stats = regionprops(S1, “Area”); if isempty(stats) areaValue = 0; else areaValue = sum([stats.Area]); end areaStats(i) = areaValue; end
%Overlay skeleton on original image	%Prune small spurs
bAnnotated = labeloverlay(I,B,Transparency = 0); imageshow(bAnnotated)	bPruned = bwskel(BW,MinBranchLength = 15); bPrunedAnnotated = labeloverlay(I,bPruned,Transparency = 0); imageshow(bPrunedAnnotated)

In this study, small spurs were not pruned before area measurement.

**Table 2:** List of primary antibodies.

Marker	Function
CD20	B lymphocytes [59]
CD79a	B lymphocytes [60]
CD3	T lymphocytes [61]
CD5	T lymphocytes and subtype of more aggressive DLBCL [62]
CD10 (MME, CALLA)	Germinal center [18,63,64,65,66,67,68,69,70]
BCL6	Germinal center [18,63,64,65,66,67,68,69,70]
MUM1 (IRF4)	Plasma cell differentiation [18,63,64,65,66,67,68,69,70,71,72]
Ki67	Cell proliferation [73,74]
MYC	Proto-oncogene [75,76]
CDK6	Cell cycle [77,78,79]
E2F1	Cell cycle, germinal center, apoptosis [69,80,81,82]
p53	Tumor-suppressor gene p53 [83,84,85,86]
LMO2	Hematopoietic development, germinal center [87,88,89]
IL-10	Immuno-oncology target [90,91,92,93]
PD-L1	Immuno-oncology target [55,94,95,96,97,98]
CSF1R	Immuno-oncology target [99,100]
CD163	Tumor-associated macrophages (TAMs) [101,102,103,104]
CASP8	Apoptosis [105,106,107]
TNFAIP8	Apoptosis [108,109,110]
BCL2	Apoptosis [111,112,113]
ISY1	Spliceosome-associated RNA-binding protein [114,115,116]
MYB	Regulation of hematopoiesis [117]
MDM2	Cell cycle [118]

**Table 3:** Clinicopathological characteristics of the series.

Variable	Frequency
Age > 60 years	77/110 (70.0%)
Male	57/110 (51.8%)
Location	
Nodal (+spleen)	57/110 (51.8%)
Waldeyer’s ring	10/110 (9.1%)
Gastrointestinal	13/110 (11.8%)
Other extranodal	30/110 (27.3%)
LDH high	63/100 (63.0%)
ECOG performance status ≥ 2	11/81 (73.6%)
International Prognostic Index (IPI)	
Low + Low-intermediate	58/87 (66.7%)
High + High-intermediate	29/87 (33.3%)
Treatment	
RCHOP	69/94 (73.4%)
RCHOP-like	20/94 (21.3%)
Others	5/94 (5.3%)
Clinical response	64/88 (72.7%)
High sIL2R	76/96 (79.2%)
Immunophenotype by IHC	
CD20	110/110 (100%)
CD3	0/110 (0%)
CD5	13/109 (11.9%)
CD10	31/109 (28.4%)
BCL2	87/109 (79.8%)
BCL6	73/109 (67.0%)
Cell of origin Hans’ classifier non-GCB	75/108 (69.4%)
*MYC* rearrangement by FISH	5/94 (5.3%)
*BCL2* rearrangement by FISH	3/93 (3.2%)
Overall survival death event	52/110 (47.3%)
Death before 2 years	37/110 (33.6%)

LDH, lactate dehydrogenase; ECOG, Eastern Cooperative Oncology Group (ECOG) Performance Status; RCHOP, rituximab, cyclophosphamide, doxorubicin, vincristine, and prednisone; sIL2R, soluble interleukin-2 receptor alpha; IHC, immunohistochemistry; FISH, fluorescence in situ hybridization.

**Table 4:** Correlation between the skeletonization area and clinical variables.

Variable	Low Skeletonization	High Skeletonization	*p* Value
Age > 60 years	29/45 (64.4%)	48/65 (73.8%)	0.300
Male	25/45 (55.6%)	32/65 (49.2%)	0.564
Location			
Nodal (+Spleen)	26/45 (57.8%)	31/65 (47.7%)	0.398
Waldeyer’s ring	2/45 (4.4%)	8/65 (12.3%)	
Gastrointestinal	4/45 (8.9%)	9/65 (13.8%)	
Other extranodal	13/45 (28.9%)	17/65 (26.2%)	
Treatment			
RCHOP	33/42 (78.6%)	36/52 (69.2%)	0.583
RCHOP-like	7/42 (16.7%)	13/52 (25.0%)	
Others	2/42 (4.8%)	3/52 (5.8%)	
LDH High	25/43 (58.1%)	38/57 (66.7%)	0.409
ECOG PS 2–4	3/37 (8.1%)	8/44 (18.2%)	0.214
Stage III–IV	18/41 (43.9%)	26/52 (50.0%)	0.676
IPI HI + H	10/38 (26.3%)	19/49 (38.8%)	0.257
Clinical response	33/41 (80.5%)	31/47 (66.0%)	0.154
High sIL2R	32/42 (76.2%)	44/54 (81.5%)	0.615
B symptoms	8/38 (21.1%)	14/45 (31.1%)	0.330
Death < 2 years	9/45 (20%)	28/65 (43.1%)	0.014

Pearson chi-square and/or Fisher’s exact test.

**Table 5:** Correlation between skeletonization and death event < 2 years of overall survival.

Variable	Low Skeletonization	High Skeletonization	*p* Value
Death < 2 years	9/45 (20%)	28/65 (43.1%)	0.014
Others	36/45 (80%)	37/65 (56.9%)	

Fisher’s exact test (exact sig. (2-sided)).

**Table 6:** Correlation between the skeletonization area and pathological variables.

Variable	Low Skeletonization	High Skeletonization	*p* Value
CD3 positive	0/45 (0.0%)	0/65 (0.0%)	0.512
CD20 positive	45/45 (100.0%)	65/65 (100.0%)	N/A
CD5 positive	10/44 (22.7%)	3/65 (4.6%)	0.006
CD10 positive	14/44 (31.8%)	17/65 (26.2%)	0.525
BCL6 positive	29/45 (64.4%)	44/64 (68.8%)	0.682
MUM1 positive	29/45 (64.4%)	44/64 (68.8%)	0.682
LMO2 high	22/44 (50%)	28/44 (63.6%)	0.282
Non-GCB	30/44 (68.2%)	45/64 (70.3%)	0.834
High Ki67	24/44 (54.5%)	33/54 (61.1%)	0.543
EBER+	4/44 (9.1%)	24/64 (37.5%)	<0.001
*BCL2* rearrangement	0/42 (0.0%)	3/51 (5.9%)	0.249
*MYC* rearrangement	1/42 (2.4%)	4/52 (7.7%)	0.376
Double-hit	0/42 (0.0%)	0/49 (0.0%)	N/A
High entropy (>7.2)	7/45 (15.6%)	17/64 (26.6%)	0.241

Pearson Chi-Square and/or Fisher’s exact test.

**Table 7:** Correlation between the skeletonization area and germinal center, cell cycle pathological, and immuno-oncology variables.

Variable	Low Skeletonization	High Skeletonization	*p* Value
Ki69	16.7 ± 15.9%	14.9 ± 13.5%	0.508
LMO2	2.2 ± 3.7%	3.1 ± 3.5%	0.109
MYC Y69	5.3 ± 4.8%	4.8 ± 5.7%	0.231
MDM2	12.4 ± 9.2%	8.9 ± 5.7%	0.083
CDK6	5.8 ± 7.4%	3.9 ± 5.7%	0.052
E2F1	2.4 ± 2.1%	1.1 ± 1.1%	<0.001
BCL2	9.4 ± 11.5%	3.6 ± 5.5%	0.010
CASP8	7.9 ± 8.9%	5.9 ± 8.2%	0.163
MYOB	25.2 ± 150.3%	24.3 ± 150.4%	0.155
TP53	5.2 ± 8.3%	5.4 ± 8.5%	0.550
cPARP	0.9 ± 1.2%	0.9 ± 1.2%	0.793
cCASP3	1.4 ± 2.3%	1.1 ± 1.2%	0.850
ISY1	2.4 ± 2.8%	1.1 ± 2.1%	0.012
TNFAIP8	48.0 ± 26.5%	34.6 ± 23.6%	0.014
CSF1R	32.9 ± 27.3%	34.5 ± 28.2%	0.675
CD163	34.1 ± 24.5%	37.4 ± 24.9%	0.372
IL-10	8.9 ± 10.8%	10.3 ± 13.1%	0.957
PD-L1	8.5 ± 11.7%	15.7 ± 18.3%	0.235

Independent-samples Mann–Whitney U test.

**Table 8:** Multivariate COX regression analysis for overall survival.

	*p* Value	HR (Exp(B))	95% CI for Exp(B)
IPI	0.113	1.70	0.88–3.29
Hans’ classifier	0.016	2.84	1.21–6.67
EBER	0.108	1.91	0.87–4.18
Skeletonization	0.030	2.21	1.08–4.53

IPI, International Prognostic Index for DLBCL prognosis.

**Table 9:** Multivariate COX regression analysis for overall survival, including entropy and skeletonization.

	*p* Value	HR (Exp(B))	95% CI for Exp(B)
Entropy	0.004	2.44	1.32–4.49
Skeletonization	0.003	2.45	1.35–4.54

**Table 10:** Multivariate COX regression analysis for overall survival, including EBER and skeletonization.

	*p* Value	HR (Exp(B))	95% CI for Exp(B)
Entropy	0.015	2.20	1.162–4.14
EBER	0.001	2.63	1.46–4.76

HR, hazard risk.

**Table 11:** Bivariate correlation between skeletonization area and germinal center, cell cycle pathological, and immuno-oncology variables.

Variable	Pearson Correlation	*p* Value
Ki69	−0.177	0.095
LMO2	−0.050	0.642
MYC Y69	−0.078	0.465
MDM2	−0.324	0.002
CDK6	−0.116	0.281
E2F1	−0.436	<0.001
BCL2	−0.352	<0.001
CASP8	−0.252	0.017
MYO	0.067	0.531
TP53	−0.016	0.878
cPARP	−0.101	0.348
cCASP3	−0.141	0.187
ISY1	−0.274	0.009
TNFAIP8	−0.216	0.042
CSF1R	0.097	0.361
CD163	0.128	0.185
IL-10	−0.006	0.955
PD-L1	0.309	0.002
Entropy	0.280	0.003

**Table 12:** Clinicopathological characteristics of the 30 cases of DLBCL with gene expression data.

Variable	Frequency
High skeletonization	18/30 (60%)
High entropy (>7.2)	6/30 (20%)
Age > 60 years	23/30 (76.7%)
Male	18/30 (60%)
Location	
Nodal (+spleen)	16/30 (53.3%)
Waldeyer’s ring	1/30 (3.3%)
Gastrointestinal	2/30 (6.7%)
Other extranodal	11/30 (36.7%)
LDH high	22/27 (81.5%)
International Prognostic Index (IPI)	
Low	6/28 (21.4%)
Low-intermediate	14/28 (50%)
High-intermediate	4/28 (14.3%)
High	4/28 (14.3%)
Treatment	
RCHOP	15/24 (62.5%)
RCHOP-like	8/24 (33.3%)
Others	1/24 (4.2%)
Clinical response	9/24 (37.5%)
High sIL2R	23/27 (85.2%)
Immunophenotype by IHC	
CD20	30/30 (100%)
CD3	0/30 (0%)
CD5	6/30 (20%)
CD10	8/30 (26.7%)
BCL2	25/30 (83.3%)
BCL6	22/30 (73.3%)
Cell of origin Lymph2Cx	
GCB	13/29 (44.8%)
ABC	9/29 (31.0%)
Unclassified	7/29 (24.1%)
Cell of origin Hans	
GCB	11/30 (36.7%)
Non-GCB	19/30 (63.3%)
EBER positive	8/29 (27.6%)
*MYC* rearrangement by FISH	4/28 (14.3%)
*BCL2* rearrangement by FISH	3/28 (10.7%)
Overall survival death event	21/30 (70%)
Death before 2 years	15/30 (50%)

LDH, lactate dehydrogenase; RCHOP, rituximab, cyclophosphamide, doxorubicin, vincristine, and prednisone; sIL2R, soluble interleukin-2 receptor alpha; IHC, immunohistochemistry; FISH, fluorescence in situ hybridization.

## Data Availability

Data is available in this article and upon request to Joaquim Carreras (joaquim.carreras@tokai.ac.jp).

## Abbreviations

The following abbreviations are used in this manuscript:

DLBCL	Diffuse large B-cell lymphoma
H&E	Hematoxylin and eosin
